# Supplementary material for: Characterization of the Antibiotic and Copper Resistance of Emergent Species of Onion-Pathogenic Burkholderia Through Genome Sequence Analysis and High-Throughput Sequencing of Differentially Enriched Random Transposon Mutants
Source: Pathogens. 2025 Feb 25;14(3):226. doi: 10.3390/pathogens14030226 (PMC11946587; doi:10.3390/pathogens14030226)
Supplement: Supplementary file 1 [file pathogens-14-00226-s001.zip › Figure_S1.pdf]

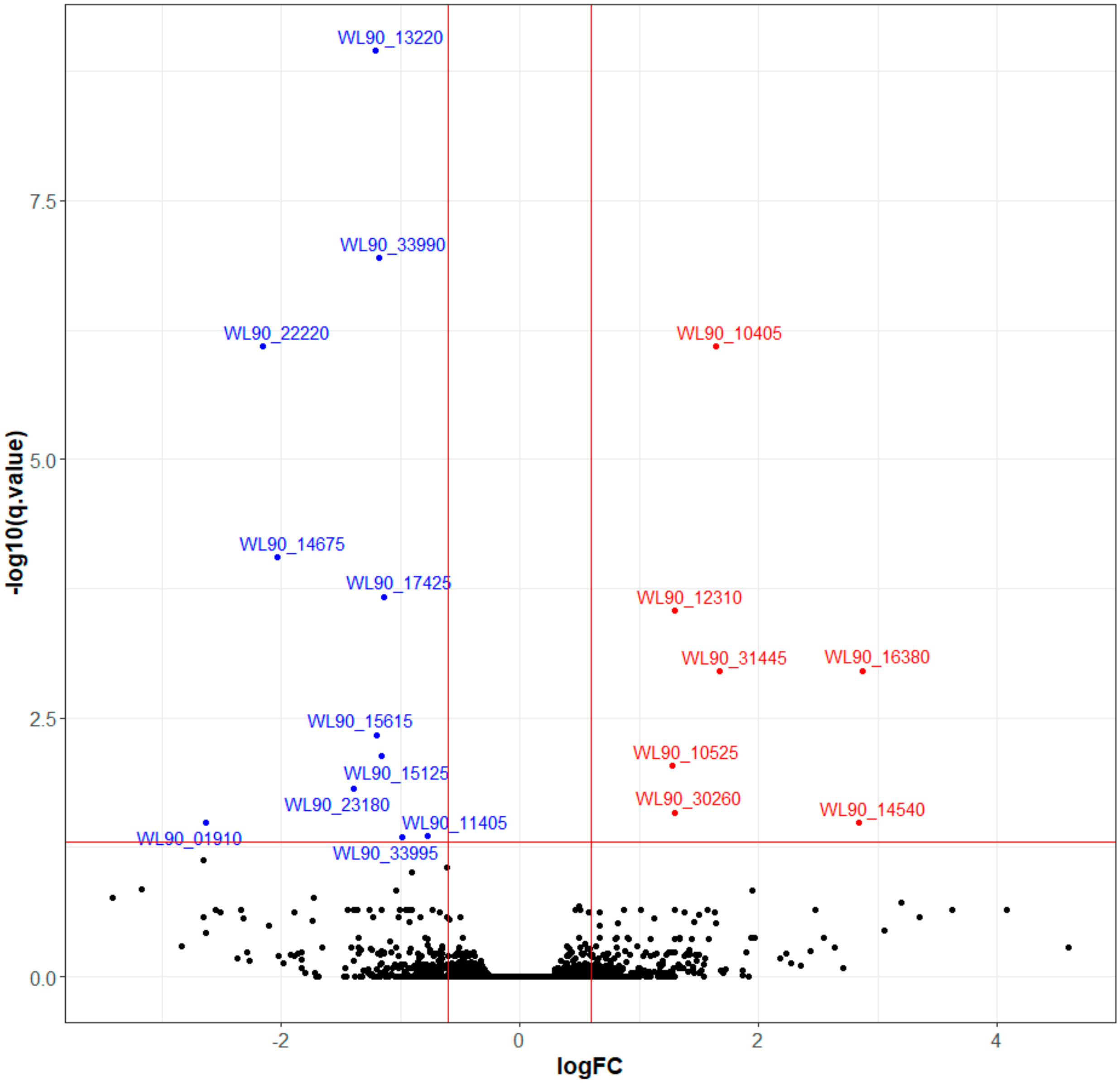

Figure S1. Genes differentially present *B. cenocepacia* CCRMBC56 mutant cells grown in the copper-stressed condition. The genes in blue and red represent the underrepresented and overrepresented genes. The figure was created using the data obtained from the conducted fitness contribution analysis.
